# Supplementary material for: Duplex DNA engagement and RPA oppositely regulate the DNA-unwinding rate of CMG helicase
Source: Nat Commun. 2020 Jul 24;11:3713. doi: 10.1038/s41467-020-17443-7 (PMC7382467; doi:10.1038/s41467-020-17443-7)
Supplement: Supplementary file 1 — Supplementary Information [file 41467_2020_17443_MOESM1_ESM.pdf]

Supplementary Information File for:

Duplex DNA engagement and RPA oppositely regulate the DNA unwinding rate of CMG helicase

Kose, et al.

Supplementary Figure 1

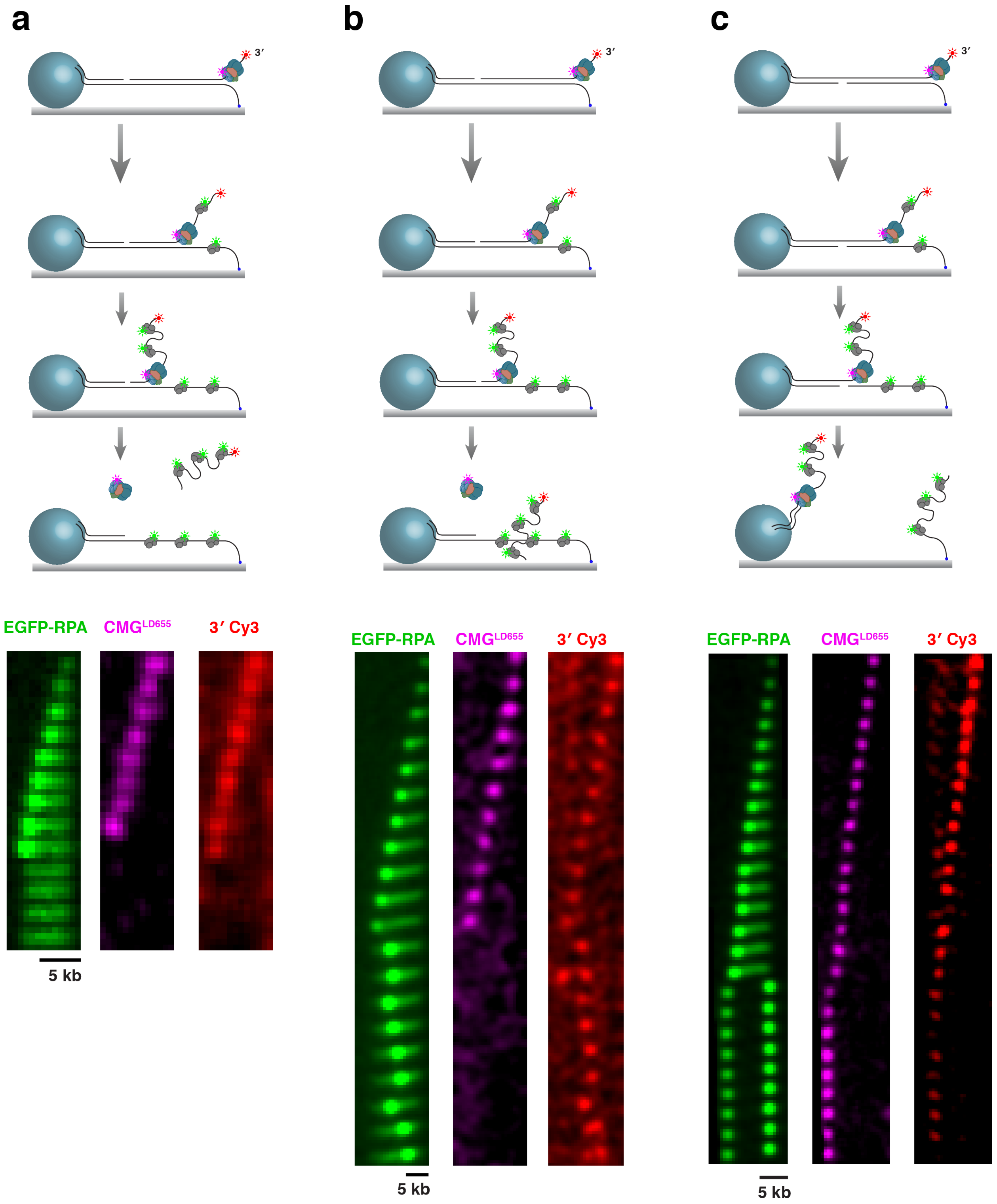

Supplementary Figure 1. Single-molecule visualization of processive DNA unwinding by CMG. a-b, Cartoon depictions of CMG meeting a nick on the leading-strand template. The leading-strand template either dissociates from (**a**) or diffuses along (**b**) the surface-immobilized DNA. Bottom images show corresponding example events. CMG does not remain on the translocation strand in **b** suggesting that it runs off the free 5′ end. **c,** CMG collides with a lagging-strand nick resulting in breakage of DNA. CMG remains on DNA. Bottom images show an example event for CMG encountering a lagging-strand nick.

Supplementary Figure 2

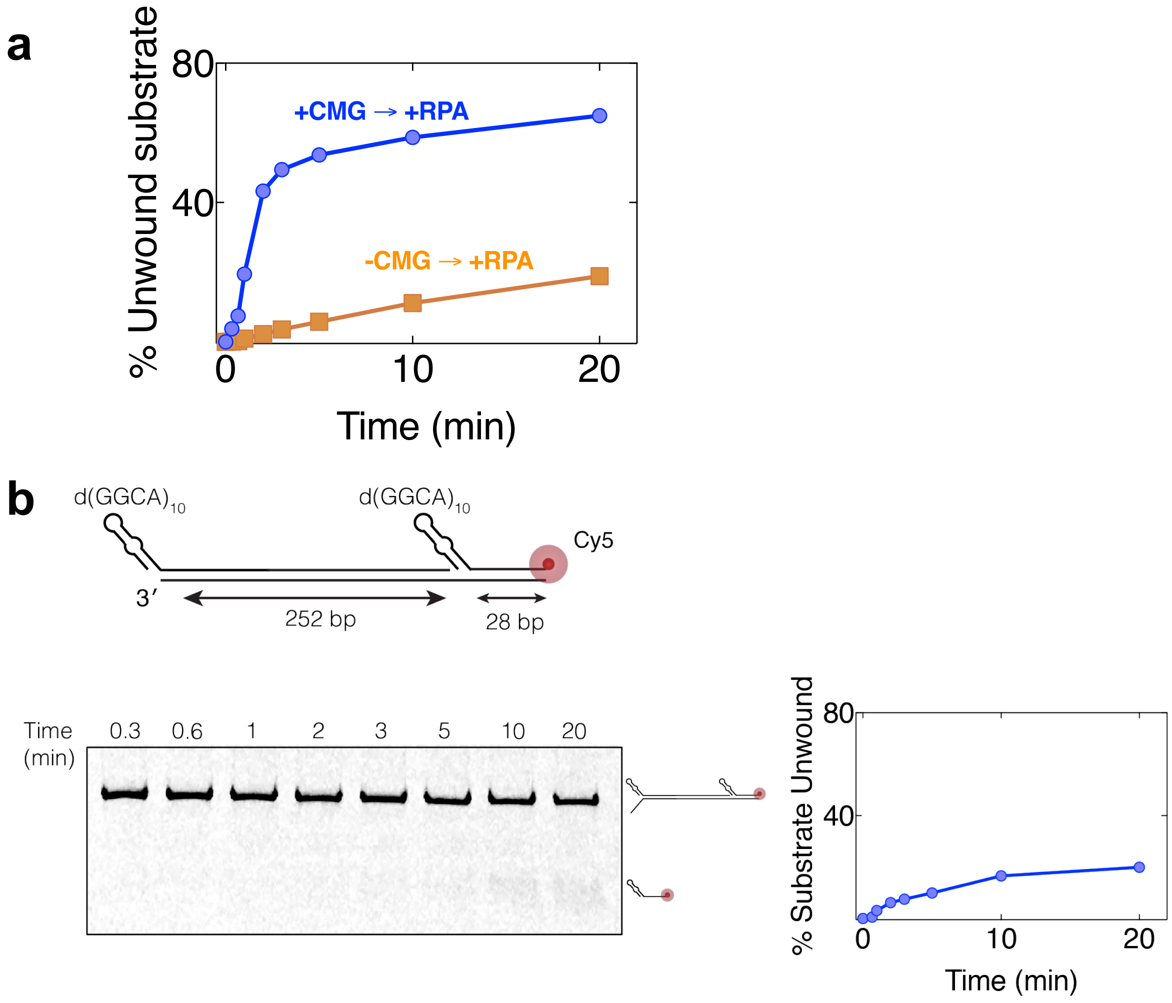

Supplementary Figure 2. Displacement of the Cy5-modified oligonucleotide downstream of 252-bp dsDNA is dependent on CMG binding to the upstream 3′ dT_{40} ssDNA. a, The same fork DNA used in Figure 2c was incubated in buffer containing or lacking CMG. RPA was then added together with ATP. RPA displaced Cy5-labelled strand to some extent even in the absence of CMG. The percentage of DNA unwound by RPA alone (-CMG→+RPA) was subtracted from that by CMG and RPA (+CMG→+RPA) to determine CMG-mediated unwinding in the presence of RPA. The data plotted in Figure 2e demonstrates measurements after this correction. **b,** The DNA substrate containing 252-bp long dsDNA, followed by 28-bp duplex and Cy5-modification on the excluded strand was incubated with CMG in the presence of ATPγS. This substrate lacked 3′ dT_{40} ssDNA overhang. Following CMG incubation with DNA, ATP and RPA were added, and the reaction was further incubated at 30°C for indicated periods of time. The right panel demonstrates the percentage of strand displacement displaced as a function of time as quantified from the gel. Cy5-modified strand was displaced to the same degree as the reaction lacking CMG in Figure S2A (-CMG→+RPA) indicating that CMG binding to 3’ dT_{40} upstream of the 252-bp dsDNA was needed for CMG-mediated unwinding. This experiments was done only once. Source data are provided as a Source Data file.

Supplementary Figure 3

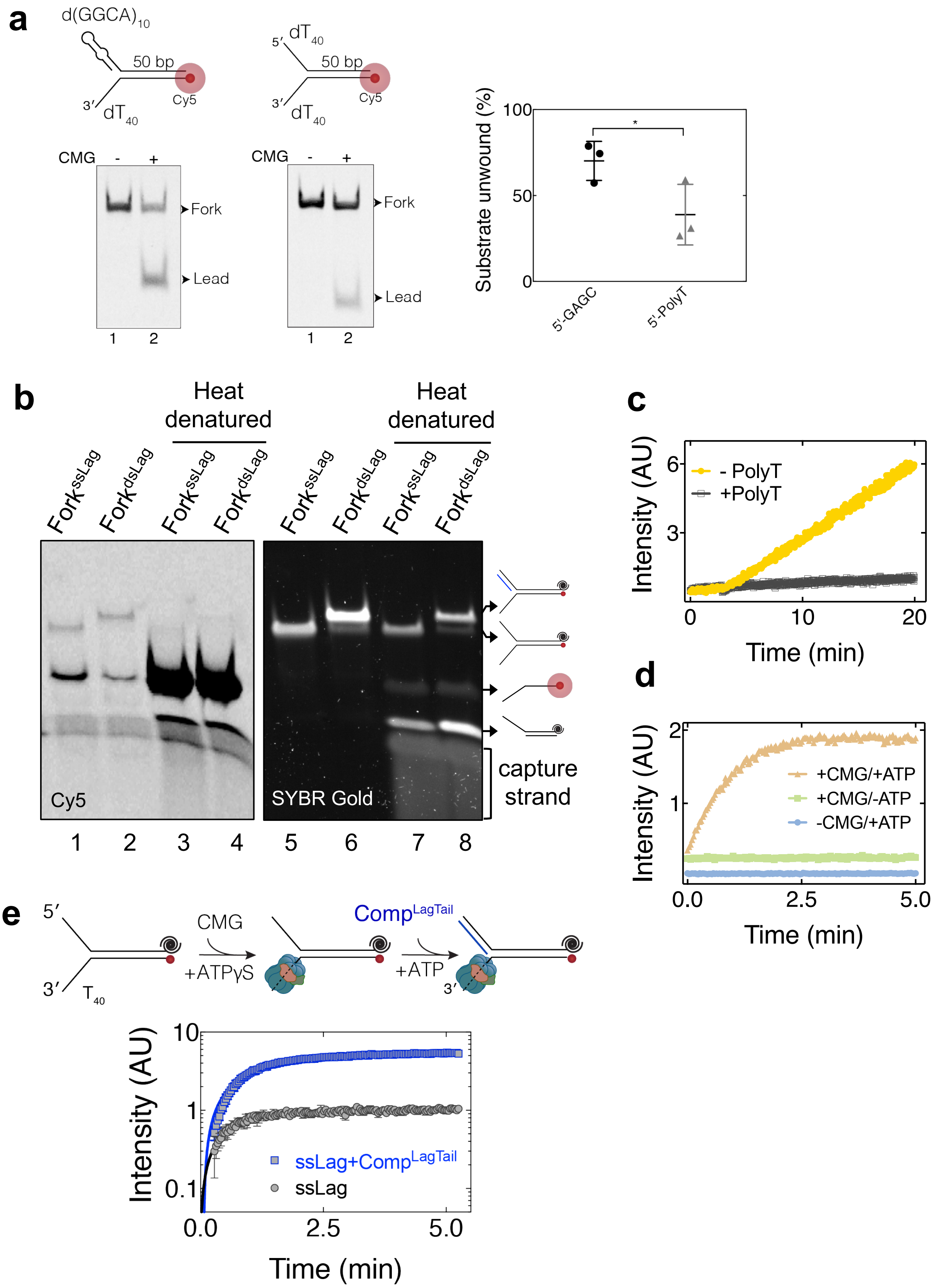

Supplementary Figure 3. The impact of the lagging-strand arm on CMG helicase activity. a, Fork DNA containing 3′ dT_{40}, 50-bp duplex, 5′ leading-strand Cy5, and either dT_{40} or $d(GAGC)_{10}$ lagging-strand arm was unwound by CMG and separated on 8% PAGE. The right panel shows quantification of DNA unwound (mean ± SD, n=3). Statistical testing; One-way ANOVA with Tukey's test. *, p=0.0357. **b,** Cy5/BHQ2-labelled fork DNA containing either single-stranded ($Fork^{ssLag}$) or double-stranded ($Fork^{dsLag}$) lagging-strand arm was separated on 8% PAGE. Fluorescence of Cy5-labelled strand is quenched and increases upon heat denaturation (compare lanes, 1-2 and 3-4). The right panel shows the same gel visualized under UV after SYBR Gold staining. A capture strand complementary to the BHQ2-modified strand was included in heat-denatured samples to prevent reannealing. This experiment was done only once. **c,** CMG was mixed with ATP and Cy5/BHQ2-labelled $Fork^{dsLag}$ in the absence (grey) or presence (yellow) of free dT_{40} oligonucleotide. Excess dT_{40} prevents CMG binding to fork DNA and subsequent unwinding. **d,** Time-dependent fluorescence intensity of Cy5/BHQ2-labelled $Fork^{dsLag}$ containing 28-bp parental duplex. Fluorescence increase is strictly dependent on the presence of CMG during ATPγS incubation as well as subsequent ATP addition. **e,** Cy5/BHQ2-labelled $Fork^{ssLag}$ was pre-incubated with CMG. ATP solution with (blue) or without (black) an oligonucleotide complementary to the 22-nt lagging-strand tail ($Comp^{LagTail}$) was added, and fluorescence intensity was measured. The data represent mean ± SD (n=3 independent experiments). Source data are provided as a Source Data file.

Supplementary Figure 4

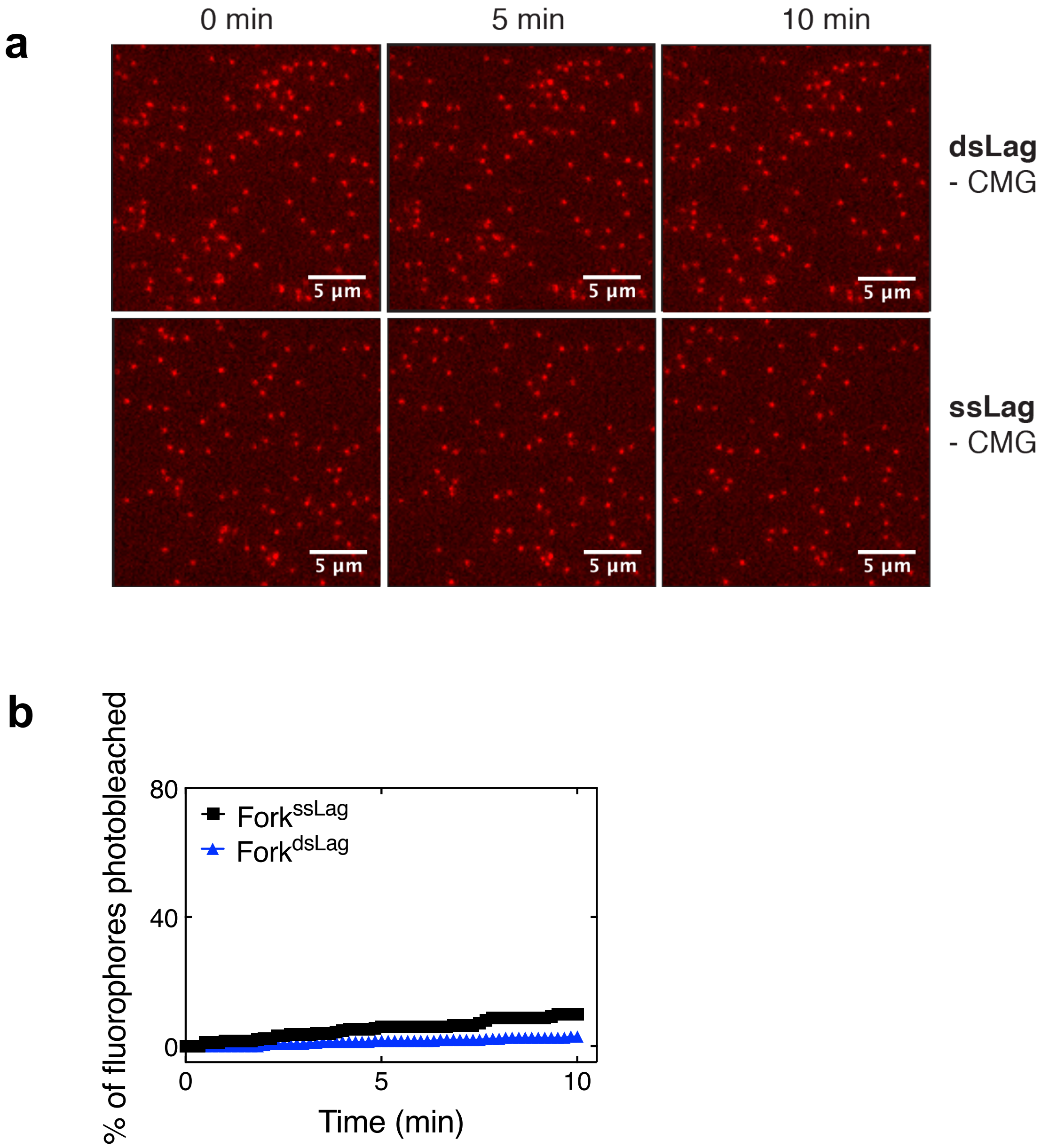

Supplementary Figure 4. Photostability of Atto647N fluorophore. a, Atto647N-labelled fork DNA was immobilized and imaged under the same experimental conditions for DNA unwinding assay (Figure 4) except CMG was omitted from ATPγS buffer. This experiment was repeated independently two times with similar results. **b,** Percentage of Atto647N spots photobleached as a function of time (Fork^{ssLag} N=251 molecules, Fork^{dsLag} N=306 molecules, each from two independent experiments). Source data are provided as a Source Data file.

Supplementary Figure 5

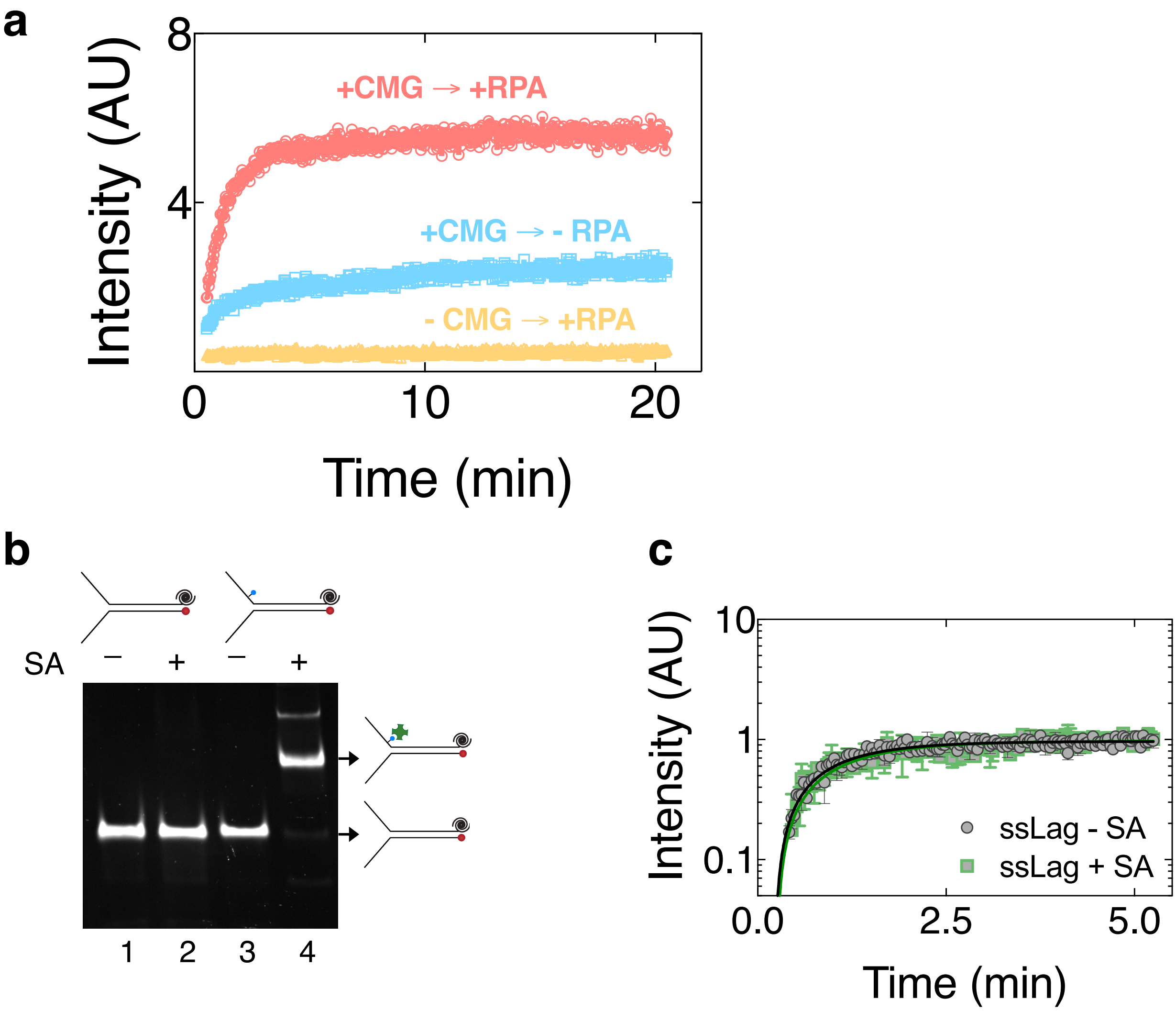

Supplementary Figure 5. Binding of a protein to the lagging-strand arm of fork DNA. a, Fork^{ssLag} was incubated with buffer containing or lacking CMG in the presence of ATPγS for 90 minutes. Buffer or RPA was subsequently added and incubated for 10 minutes. ATP was then added to initiate CMG translocation. A competitor oligonucleotide was included with ATP addition to prevent further RPA and CMG binding during unwinding. When CMG was pre-bound to the fork, addition of RPA stimulated unwinding (compare +CMG→+RPA to +CMG→-RPA) similar to shown in Figure 5. Importantly, when CMG was omitted from the reaction, addition of RPA did not lead to detectable unwinding (-CMG→+RPA). **b,** Binding of streptavidin (SA) to the lagging-strand biotin on fork DNA is monitored by separating DNA on 8% PAGE and subsequent staining with SYBR Gold. Addition of streptavidin to biotin-modified fork leads to mobility shift (lane 4). This experiment was done only once. **c,** CMG-catalyzed single turn-over unwinding of fork DNA lacking a biotin on the lagging-strand arm in the absence (black) and presence (green) of streptavidin (SA). Data represent mean ± SD (n=3 independent experiments). Source data are provided as a Source Data file.

Supplementary Figure 6

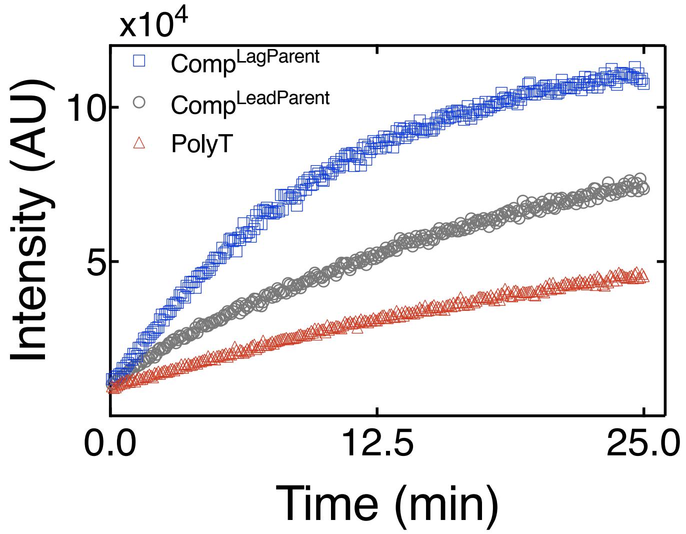

Supplementary Figure 6. The impact of Comp^{LeadParent} on DNA unwinding activity by CMG. Single turn-over unwinding of fork DNA used in Figure 6b containing 60-bp duplex region and hairpin lagging-strand arm. CMG was bound to the fork in the presence of ATPγS. Subsequently ATP was added and unwinding was monitored through Cy5 fluorescence. An oligonucleotide complementary to either the lagging- (Comp^{LagParent}) or the leading-template (Comp^{LeadParent}) within the duplex region or 40-nt long polyT oligonucleotide was added with ATP. Source data are provided as a Source Data file.

Supplementary Figure 7

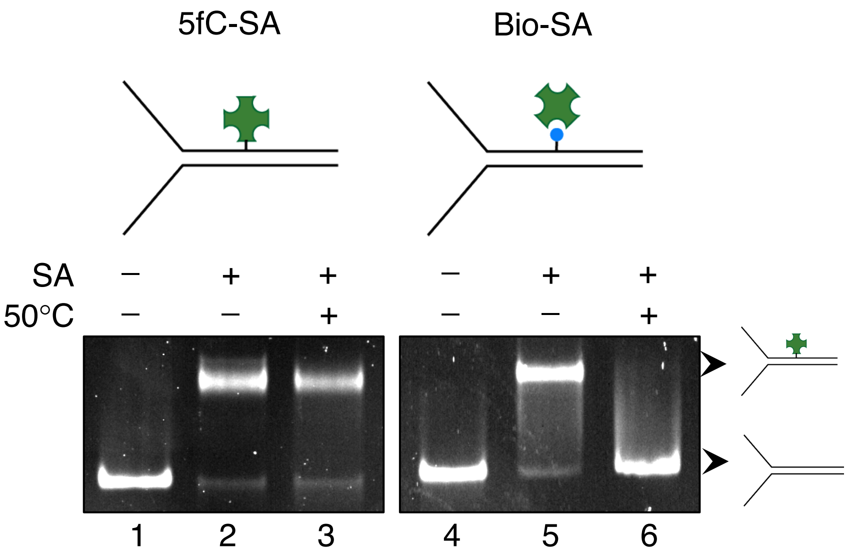

Supplementary Figure 7. 5-Formyl-cystosine-streptavidin crosslinked fork DNA substrate. Fork DNA containing either 5-formyl-cytosine-streptavidin (5fC-SA) crosslink (lane 2) or biotin-streptavidin (bio-SA) complex (lane 4) was heated to 50°C for 10 minutes in the presence of 1 μM 5fC-modified oligonucleotide or 1 μM free biotin, respectively, and separated on 8% PAGE. While streptavidin dissociated from biotin upon heat treatment (lane 6), 5fC-SA crosslink remained stable (lane 3). Source data are provided as a Source Data file.

Supplementary Table 1. Oligonucleotides used in each DNA substrate.

Substrate Name	Oligonucleotides Used	Associated Figures
236-bp fork template (internally Cy5 labelled)	Oligo-2, Oligo-3, Oligo-4, Oligo-5	Fig. 2a
252-bp fork template (3′-Cy5)	Oligo-2, Oligo-17, Oligo-4, Oligo-5, Oligo-15, Oligo-16, Oligo-Cy5-6	Fig. 2c-d, Supp. Fig. 2a
252-bp fork template lacking 3′-PolyT tail	Oligo-2, Oligo-3, Oligo-4, Oligo-18, Oligo-15, Oligo-16, Oligo-Cy5-6	Supp. Fig. 2b
$Fork^{ssLag}$	Oligo-BHQ2-2, Oligo-Cy5-4	Fig. 3, 5a, 6a, Supp. Fig. 3e
$Fork^{dsLag}$	Oligo-12, Oligo-BHQ2-2, Oligo-Cy5-4	Fig. 3, 5b, Supp. Fig. 3b-d
$Fork^{ssLag}$-Bio	Oligo-BHQ2-Bio, Oligo-Cy5-4	Fig. 5c, Supp. Fig. 5b
Fork-5′-PolyT	Oligo-Bio-2, Oligo-Cy5-5	Supp. Fig. 3a
Fork-5′-GAGC	Oligo-Bio-1, Oligo-Cy5-5	Supp. Fig. 3a
$Fork^{ssLag}$-TIRF	Oligo-Atto, Oligo-19, Oligo-Bio-5	Fig. 4, Supp. Fig. 4
$Fork^{dsLag}$-TIRF	Oligo-Atto, Oligo-19, Oligo-Bio-5, Oligo-12	Fig. 4, Supp. Fig. 4
60-bp fork with 5′-GAGC	Oligo-11, Oligo-BHQ2-1, Oligo-Cy5-1	Fig. 6b
Fork (5fC-SA crosslink)	Oligo-5fC-1, Oligo-9, Oligo-14, Oligo-IBRQ	Fig. 7
Fork (no crosslink)	Oligo-7, Oligo-14, Oligo-Cy5-2, Oligo-IBRQ	Fig. 7
Fork-Bio-SA	Oligo-10, Oligo-Bio-3, Oligo-Cy5-3	Supp. Fig. 6
10kb-substrate (TIRF)	pUC19 as the PCR template, Oligo-Bio-4, Oligo-Cy3, Oligo-1, Oligo-8	Fig. 1, Supp. Fig. 1

Supplementary Table 2. Sequences of oligonucleotides used to prepare DNA substrates.

Oligo-1	ATGCCGGGAGCAGACAAGCCCGTC
Oligo-2	CCTCCAAAAAAGCCTCCTCACTA
Oligo-3	CTGGCTTAACTATGCGGCATCAG
Oligo-4	[5'-Phos]GGCAGGCAGGCAGGCAGGCAGGCAGGCAGGCAGGCAGGTTGGCGAATTCCCATTGCC
Oligo-5	CATGGGCAATGGGAATTCGCCAACCTTTTTTTTTTTTTTTTTTTTTTTTTTTTTTTTTTTTTTTTTT
Oligo-6	TTTTTTTTTTTTTTTTTTTTTTTTTTTTTTTTTTTTTTTT
Oligo-7	GGCAGGCAGGCAGGCAGGCAGGCAGGCAGGCAGGCAGGTTGGCGAATTC
Oligo-8	ATGGGCCCAGCTGGCACGACAGGTTTCCCG
Oligo-9	GGCAGGCAGGCAGGCAGGCAGGCAGGCAGGCAGGCAGGTTGGCGAATTCCCATTG
Oligo-10	GCTCGTTTTACAACGTCGTGCTGAGGTACCGGATGCTGAGGCAATGGGAATTCGCCAACCTTTTTTTTTTTTTTTTTTTTTTTTTTTTTTTTTTTTTTTT
Oligo-11	GGCAGGCAGGCAGGCAGGCAGGCAGGCAGGCAGGCAGGTTGGCGAATTCCCATTGCCTCAGCATCCGGTACC
Oligo-12	CGCTGTCTGTTCCCTTCTTGTC
Oligo-13	GCTCTTTGTTCCTT
Oligo-14	[5'-Phos]TCGCCAACCTTTTTTTTTTTTTTTTTTTTTTTTTTTTTTTTTTTTTTTT
Oligo-15	[5'-Phos]AGCTACTCCAGCGGCGGG
Oligo-16	GCTCTTTGTTCCTTCTCCTGTCCTTCCTTTCCCGCCGCTGGAGT
Oligo-17	TCCTAAGCTTACTGGCTTAACTATGCGGCATCAG
Oligo-18	CATGGGCAATGGGAATTCGCCAACCTTTTTTTTTTTTTTTTTTTTTTTTTTTTTTTTTTTTTTTTTT
Oligo-19	CATGCCACACACCGAGACACAC
Oligo-20	CCGGATGCTGAGGCAATGGGAATTCGCCAACC
Oligo-21	AGGAAGGACAGGAGAAGGAACAAAGAGC
Oligo-22	GGTTGGCGAATTCCCATTGCCTCAGCATC
Oligo-BHQ2-1	[5'-Phos]TCAGCACGACGTTGTAAAACGAGC[3'-BHQ2]
Oligo-BHQ2-2	GACAAGAAGGGAACAGACAGCGAGGAAGGACAGGAGAAGGAACAAAGAGC[3'-BHQ2]
Oligo-BHQ2-Bio	GACAAGAAGGGAACAGA[Biotin-dT]AGCGAGGAAGGACAGGAGAAGGAACAAAGAGC[3'-BHQ2]

Oligo-Bio-1	TTTTTTTTTTTTTTTTTTTTTTTTTTTTTTTTTTTTTTTTGACGCTGCCGAATTCTGGCTTGC[Biotin-dT]AGGACATTACAGGATCGTTCGGTCTC
Oligo-Bio-2	GGCAGGCAGGCAGGCAGGCAGGCAGGCAGGCAGGCAGGCAGACGCTGCCGAATTCTGGCTTGC[Biotin-dT]AGGACATTACAGGATCGTTCGGTCTC
Oligo-Bio-3	GGCAGGCAGGCAGGCAGGCAGGCAGGCAGGCAGGCAGGCAGGTTGGCGAATTCCCATTGCCTCAGCA[Biotin-dT]CCGGTACC
Oligo-Bio-4	[5'-BiotinTEG]GACAAGAAGGGAACAGACAGCGAGGAAGGACAGGAGAAGGAACAAAGAGC
Oligo-Bio-5	[5'-BiotinTEG]ATTCGTCTGCCTGCTTCGTCGTCTCTTGTGTGTCTCGGTGTGTGGCATGGCTCTTTGTTCCTTCTCCTGTCCTTCCTTTTTTTTTTTTTTTTTTTTTTTTTTTTTTTTTTTTTTTTTTT
Oligo-Cy3	[5'-Phos]AGGTCGCCGCCCGCTCTTTGTTCCTTCTCCTGTCCTTCCTTTTTTTTTT[Cy3-dT]TTTTTTTTTTTTTTTTTTTTTTTTTTTTTTTTTTTTTTTT
Oligo-Cy5-1	[5'-Cy5]GCTCGTTTTACAACGTCGTGCTGAGGTACCGGATGCTGAGGCAATGGGAATTCGCCAACCTTTTTTTTTTTTTTTTTTTTTTTTTTTTTTTTTTTTTTTT
Oligo-Cy5-2	[5'-Phos]CCATTGCCTCAGCATCCGGTACCTCAGCACGACGTTGTAAAACGAGC[3'-Cy5]
Oligo-Cy5-3	[5'-Phos]TCAGCACGACGTTGTAAAACGAGC[3'-Cy5]
Oligo-Cy5-4	[5'-Cy5]GCTCTTTGTTCCTTCTCCTGTCCTTCCTTTTTTTTTTTTTTTTTTTTTTTTTTTTTTTTTTTTTTTT
Oligo-Cy5-5	[5'-Cy5]GAGACCGAACGATCCTGTAATGTCCTAGCAAGCCAGAATTCGGCAGCGTCTTTTTTTTTTTTTTTTTTTTTTTTTTTTTTTTTTTTTTTT
Oligo-Cy5-6	GGCAGGCAGGCAGGCAGGCAGGCAGGCAGGCAGGCAGGCAGGAAGGACAGGAGAAGGAACAAAGAGC[3'-Cy5]
Oligo-5fC	[5'-Phos]CCTCAGCAT[5Formyl-dC]CGGTACCTCAGCACGACGTTGTAAAACGAGC[3'-Cy5]
Oligo-IBRQ	[5'-Iowa Black RQ]GCTCGTTTTACAACGTCGTGCTGAGGTACCGGATGCTGAGGCAATGGGAAT
Oligo-Atto	[5'-Atto647N]-TGACAAGAAGGGAACAGACAGCGAGGAAGGACAGGAGAAGGAACAAAGAGC
