# Supplementary material for: Duplex DNA engagement and RPA oppositely regulate the DNA-unwinding rate of CMG helicase
Source: Nat Commun. 2020 Jul 24;11:3713. doi: 10.1038/s41467-020-17443-7 (PMC7382467; doi:10.1038/s41467-020-17443-7)
Supplement: Supplementary file 3 — Reporting Summary [file 41467_2020_17443_MOESM3_ESM.pdf]

nature research

Corresponding author(s): Hasan Yardimci

Last updated by author(s): 6/10/2020

Reporting Summary

Nature Research wishes to improve the reproducibility of the work that we publish. This form provides structure for consistency and transparency in reporting. For further information on Nature Research policies, see Authors & Referees and the Editorial Policy Checklist.

Statistics

For all statistical analyses, confirm that the following items are present in the figure legend, table legend, main text, or Methods section.

n/a	Confirmed	
☐	☒	The exact sample size (*n*) for each experimental group/condition, given as a discrete number and unit of measurement
☐	☒	A statement on whether measurements were taken from distinct samples or whether the same sample was measured repeatedly
☐	☒	The statistical test(s) used AND whether they are one- or two-sided<br>*Only common tests should be described solely by name; describe more complex techniques in the Methods section.*
☒	☐	A description of all covariates tested
☒	☐	A description of any assumptions or corrections, such as tests of normality and adjustment for multiple comparisons
☐	☒	A full description of the statistical parameters including central tendency (e.g. means) or other basic estimates (e.g. regression coefficient) AND variation (e.g. standard deviation) or associated estimates of uncertainty (e.g. confidence intervals)
☐	☒	For null hypothesis testing, the test statistic (e.g. *F*, *t*, *r*) with confidence intervals, effect sizes, degrees of freedom and *P* value noted<br>*Give P values as exact values whenever suitable.*
☒	☐	For Bayesian analysis, information on the choice of priors and Markov chain Monte Carlo settings
☒	☐	For hierarchical and complex designs, identification of the appropriate level for tests and full reporting of outcomes
☒	☐	Estimates of effect sizes (e.g. Cohen's *d*, Pearson's *r*), indicating how they were calculated

Our web collection on statistics for biologists contains articles on many of the points above.

Software and code

Policy information about availability of computer code

Data collection	PHERAStar FS (BMG Lab Tech)<br>NIS-Elements AR version 4.51.01
Data analysis	NIS-Elements AR Analysis version 4.51.01<br>Prism 7 GraphPad Software, La Jolla, CA, USA<br>Fiji (ImageJ) version 1.0

For manuscripts utilizing custom algorithms or software that are central to the research but not yet described in published literature, software must be made available to editors/reviewers. We strongly encourage code deposition in a community repository (e.g. GitHub). See the Nature Research guidelines for submitting code & software for further information.

Data

Policy information about availability of data

All manuscripts must include a data availability statement. This statement should provide the following information, where applicable:

- Accession codes, unique identifiers, or web links for publicly available datasets
- A list of figures that have associated raw data
- A description of any restrictions on data availability

The source data underlying Figs. 1e, 2a, c-f, 3b-e, 4c, 5, 6, 7b and Supplementary Figs. 2, 3, 4b, 5, 6, 7 are provided as a Source Data file. Data supporting the findings of this study are available from the corresponding author upon reasonable request.

Field-specific reporting

Please select the one below that is the best fit for your research. If you are not sure, read the appropriate sections before making your selection.

☒ Life sciences ☐ Behavioural & social sciences ☐ Ecological, evolutionary & environmental sciences

For a reference copy of the document with all sections, see nature.com/documents/nr-reporting-summary-flat.pdf

Life sciences study design

All studies must disclose on these points even when the disclosure is negative.

Sample size	This study involves biochemical assays as well as single-molecule imaging data that include different number of molecules in each dataset.
Data exclusions	No data was excluded throughout the study.
Replication	Multiple replicates of the same experiments were performed. Multiple molecules were observed in single-molecule experiments. Details can be found in the manuscript. All attempts of replication was successful.
Randomization	This study does not involve any patient, participant or group of people where randomisation would be essential to avoid any kind of bias.
Blinding	This study does not involve any patient, participant or group of people where blinding would be essential to avoid any kind of bias.

Reporting for specific materials, systems and methods

We require information from authors about some types of materials, experimental systems and methods used in many studies. Here, indicate whether each material, system or method listed is relevant to your study. If you are not sure if a list item applies to your research, read the appropriate section before selecting a response.

Materials & experimental systems

n/a	Involved in the study	
☐	☒	Antibodies
☐	☒	Eukaryotic cell lines
☒	☐	Palaeontology
☒	☐	Animals and other organisms
☒	☐	Human research participants
☒	☐	Clinical data

Methods

n/a	Involved in the study	
☒	☐	ChIP-seq
☒	☐	Flow cytometry
☒	☐	MRI-based neuroimaging

Antibodies

Antibodies used	PAb419 (Cell Services, STP, The Francis Crick Institute), Polyclonal anti-dig antibody (Roche, Cat no: 11333089001, LOT no: 20170100)
Validation	Polyclonal anti-dig antibody used in other studies:<br>1) Yang D, Ward A, Halvorsen K, Wong WP. Multiplexed single-molecule force spectroscopy using a centrifuge. Nat Commun. 2016;7:11026. Published 2016 Mar 17. doi:10.1038/ncomms11026<br>2) Wang W, Jossin Y, Chai G, Lien WH, Tissir F, Goffinet AM. Feedback regulation of apical progenitor fate by immature neurons through Wnt7-Celsr3-Fzd3 signalling. Nat Commun. 2016;7:10936. Published 2016 Mar 4. doi:10.1038/ncomms10936

Eukaryotic cell lines

Policy information about cell lines

Cell line source(s)	Sf9 insect cells (Thermo Fisher); High Five insect cells (Thermo Fisher)
Authentication	None. Commercial reagent for baculoviral expression.
Mycoplasma contamination	Tested by Francis Crick Institute Cell Services. We confirm that all cell lines have been tested negative for mycoplasma contamination.
Commonly misidentified lines (See ICLAC register)	No commonly misidentified cell lines were used in the study.
